# Quantitative X-ray Channel-Cut Crystal Diffraction Wavefront Metrology Using the Speckle Scanning Technique

**DOI:** 10.3390/s20226660

**Published:** 2020-11-20

**Authors:** Lian Xue, Hongxin Luo, Qianshun Diao, Fugui Yang, Jie Wang, Zhongliang Li

**Affiliations:** 1 Shanghai Synchrotron Radiation Facility, Shanghai Advanced Research Institute, Chinese Academy of Sciences, Shanghai 201800, China; xuelian@zjlab.org.cn (L.X.); luohongxin@zjlab.org.cn (H.L.); wangjie@zjlab.org.cn (J.W.); 2 Beijing Synchrotron Radiation Facility, Institute of High Energy Physics, Chinese Academy of Sciences, Beijing 100049, China; diaoqs@ihep.ac.cn (Q.D.); yangfg@ihep.ac.cn (F.Y.); 3 University of Chinese Academy of Sciences, Chinese Academy of Sciences, Beijing 100049, China

**Keywords:** X-ray crystal diffraction wavefront, channel-cut crystal, speckle-based method

## Abstract

A speckle-based method for the X-ray crystal diffraction wavefront measurement is implemented, and the slope errors of channel-cut crystals with different surface characteristics are measured. The method uses a speckle scanning technique generated by a scattering membrane translated using a piezo motor to infer the deflection of X-rays from the crystals. The method provides a high angular sensitivity of the channel-cut crystal slopes in both the tangential and sagittal directions. The experimental results show that the slope error of different cutting and etching processes ranges from 0.25 to 2.98 μrad. Furthermore, the results of wavefront deformation are brought into the beamline for simulation. This method opens up possibilities for new high-resolution applications for X-ray crystal diffraction wavefront measurement and provides feedback to crystal manufacturers to improve channel-cut fabrication.

## 1. Introduction

With the rapid development of accelerator technology, higher-quality X-ray beams can be generated from third- and fourth-generation synchrotron radiation facilities based on diffraction limit storage rings. While the high-quality X-ray beam significantly benefits the development of X-ray applications, X-ray optical transmission systems also face technical challenges. Optical elements in the transmission system are expected to provide higher flux, lower scattering, and both improved energy and spatial resolution of X-ray beams. As the photon beam from the source has a certain spectral width, it is very important to monochrome the beam before delivering it to an endstation. Therefore, the monochromator is an almost irreplaceable piece of equipment in the synchrotron beamline. A crystal monochromator is usually applied in the hard X-ray range, because the lattice constant of the crystal is similar to the wavelength of an X-ray in this range. In general, a double crystal monochromator (DCM) and channel-cut crystal monochromator (CCM) are two typical crystal monochromators in the hard X-ray range. The basic principle of these monochromators is Bragg diffraction from high-quality crystals. Structurally, a DCM is composed of two independent crystal blocks. Thus, in order to achieve a perfect decoupling between the relative positions of the first and second crystals during Bragg-angle rotation, the support of the two crystal has to be carefully designed [1]. However, CCM is simply cut out of a groove on the same crystal, which is a natural advantage for this monochromator. Both “first” and “second” crystals are fabricated from the same single piece of crystal (usually silicon), which ensures parallelism between the diffraction planes. It is unnecessary to apply an additional mechanical structure to maintain the alignment [2]. No matter what kind of monochromator, it is difficult to obtain a perfect crystal. Unfortunately, the wavefront or coherence of the transmitted beam becomes damaged upon double reflection due to surface or bulk imperfections. Especially for channel cut crystals, the difficulties of processing are mainly restricted by the processing of its inner surface. The gap between the two crystal planes is typically only 5–20 mm. It is difficult to finish the subsequent grinding and polishing of the inner surface of the crystal in such a narrow space. Paradoxically, a CCM can offer the best mechanical stability but distorts the beam at the same time due to its rough and wavy surface. Therefore, limited by processing technology, the application of channel-cut crystal monochromators has been traditionally confined to cases that can tolerate the rough surface quality from wet etching but without polishing. With the development of mechanical and polishing technology, significant improvements have been made in the fabrication of diffraction-limited X-ray optics used to pursue an aberration-free wavefront [3]. The surface of the crystal used in the CCM is also improving due to technological progress. Accordingly, it is urgent and vitally important to develop suitable metrology tools for fully characterizing the optical surface. However, even the surface profile is difficult to detect due to the special structure, let alone the influence of the diffraction surface on the wavefront. Additionally, an important cause of material distortion and wavefront aberration is the heat-load accumulated through photon absorption, even despite the cooling system.

The suitable metrology tools for channel cut crystals are at-wavelength metrology, which is one of the metrologies of optical elements. Another important branch of optical metrology is visible-light metrology. The difference between these two branches is not only the wavelength of the source used for metrology, but also the working condition of the tested optical elements. Traditionally, visible-light metrology technology primarily includes Fizeau interferometry, long-trace profilometry, and nanometer optical metrology (NOM). These technologies are routinely adopted for ex-situ measurement [4,5] of the surface profiles of a reflection type mirror with height accuracy in nanometers. Both slope and shape errors of mirrors, including planar or curved types, can be characterized over spatial frequencies ranging from 1 mm^−1^ to 1 m^−1^. Unfortunately, the application of visible-light metrology is limited to reflection type optical elements, and it is difficult to expand applications to transmission or diffractive type optical elements, such as X-ray compound refractive lenses (CRL) and crystals, respectively. As mentioned above, with results obtained by ex-situ methods, it is difficult to reflect the real performance of the tested optics under actual working conditions. To overcome these limitations, at-wavelength metrology methods have been rapidly developed over the last decade due to in-situ, high sensitivity, and at-wavelength measurements. Furthermore, the measurement resolution of visible light is approaching the diffraction limit of visible light, which limits the final measurement accuracy by ex-situ methods. However, the theoretical measurement resolution can be pushed to even lower values, since the wavelength of X-ray is much shorter than that of visible light. Of the different at-wavelength methods, Hartmann sensors [6], grating interferometers [7,8,9], and the speckle-based method [10,11,12] have been widely adapted to synchrotron and free-electron laser (FEL) facilities all over the world. Although significant developments have been made, dedicated and complex optics are still required with Hartmann and grating techniques, which limits their feasibility for widespread application.

Speckle is a well-known phenomenon within the visible spectrum [13] and can be considered as a mature field of optics that has proven highly useful in a range of scientific disciplines, including metrology, astronomy, and speckle imaging [14,15,16]. This technique was introduced only 10 years ago into the X-ray community with the technique of X-ray photon correlation spectroscopy (XPCS). When a scattering diffuser composed of small objects is placed into a fully or partially coherent beam, a near-field speckle pattern can be recorded using a suitable high spatial resolution detector. In recent years, X-ray near-field speckle methods [17,18,19] have been developed for X-ray optic characterization. The advantage of the speckle technique lies in its low requirement for coherence and in the simplicity of the wavefront modulator, such as a piece of sandpaper or a biological filter. The wavefront and associated aberrations can be measured precisely using the speckle-based technique, which has been proven applicable at both synchrotron facilities [20,21] and laboratory X-ray tube sources [22,23,24]. The speckle-based technique can be realized in two ways: X-ray speckle tracking (XST) [17] and X-ray speckle scanning (XSS) [24]. A near-field speckle pattern can be generated using a random phase modulator in any configuration. In the XST method, correlation analysis is used to compare the speckle patterns obtained with and without an object. Phase gradient information of the sample can be generated, moreover, on absorption and scattering information in two dimensions. In the XSS method, a higher spatial resolution can be obtained by scanning the phase modulator across the beam, because subsets containing more pixels are used for correlation. Normally, the first derivative (slope) of the wavefront phase is measured in the XST method, while the second derivative (curvature) of the wavefront phase is measured in the XSS method. The XSS method is more suitable for cases in which the optical element is fixed, but the surface performance of the tested optical element cannot be measured separately, because the result includes contributions from both the upstream beam and the tested optics. By contrast, in the XST method, the tested optics have to be moved out of the beam path to measure the surface directly without the influence of the upstream beam.

The near-field speckle technique is a good choice for measuring the wavefront slope error of the CCM due to its low requirements of beam coherent and experimental equipment. In addition, the effects of crystal lattice deformations, and not only those of the crystal surface shape, can be measured. In this study, the wavefront slope error caused by the channel-cut diffractive surface was measured. The speckle was generated by a scattering membrane driven by a piezo motor. This technology combines the advantages of the XST and XSS methods by moving the CCM and scanning scatterers. A nano-radian order sensitivity [25] can be achieved in the measurement of crystal slopes in both the tangential and sagittal directions. A total of five channel-cut crystals with different surface processes are investigated in this study.

## 2. Principles

**Figure 1:**
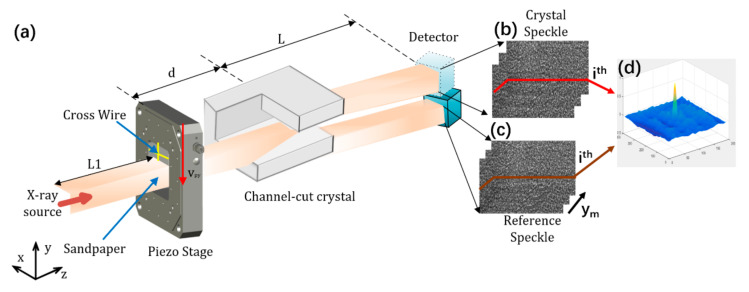
Experimental arrangement and correlation principle for absolute metrology technique. (**a**) Speckle set-up for measuring channel cuts. Stacks of crystal (**b**) and reference (**c**) speckle images retrieved by the detector. (**d**) Cross-correlation map of the two patterns shown in (**b**,**c**).

The speckle-based X-ray crystal diffraction wavefront measurement technology proposed in this study is referred to as absolute metrology technology, which has been successfully used to characterize reflective optical elements such as X-ray mirrors [26]. The principle of this technique was developed on the basis of the XST method. Absolute metrology technology is a kind of deflection angle technology. It depends on the measurement of the local wavefront gradient ∇W or phase gradient ∇ϕ through the deflection angle *α* = ∇W [17]. This method is usually more suitable for uniform samples with a slow change of optical index, in which there is no sharp edge, which allows for a high spatial resolution to be obtained. The device only needs a random phase object and a two-dimensional detector to distinguish the high spatial frequency features contained in the object. Figure 1a shows a schematic diagram of this method in the application of a channel-cut wavefront measurement. A scatterer with a random phasor is placed in a partially coherent X-ray beam. A solid film (such as sandpaper) is usually chosen to produce a static random intensity pattern (speckle), based on a variety of advantages such as ease of alignment and low sensitivity to vibration. The sandpaper is scanned by a piezo stage perpendicularly to the beam with a step size *s*. As shown in Figure 1b,c, speckle images are recorded at each sandpaper’s position *y*, and then two subsets with the *i*th rows of all the speckle images are built up. In this method, two sets of collected images are coupling together, one with crystals present in the X-ray beam (crystal speckles), and the other with crystals removed from the beam (reference speckles). A digital image correlation algorithm (DIC) with subpixel accuracy is then used to track the X-ray path [27]. The result of the operation is a correlation graph whose maximum peak position represents the displacement vector v between the two arrays $\nu =\left(ii^{\prime }\right)$. The zero-normalized cross correlation (ZNCC) [28] is used to evaluate a similarity factor between a subset of M points in the reference image *f* and a target subset centered on ($x_{0},y_{0}$) in a target image *g*.

(1) $$i^{\prime }=\max C\left(x_{0},y_{0}\right)=\max\left\{\sum_{x=-M}^{M}\sum_{y=-M}^{M}\left[\frac{\left[f\left(x,y\right)-\bar{f}\right]\left[g\left(x^{\prime },y^{\prime }\right)-\bar{g}\right]}{\Delta f\Delta g}\right]\right\}$$

Here, $\bar{f}$ and $\bar{g}$ are the mean values of the subsets, and Δf and Δg represent their respective standard deviations.

The relation between the position of the region with $\left(x_{i}^{\prime },y_{i}^{\prime }\right)$ and without $\left(x_{i},y_{i}\right)$ the crystal can be written as follows:

(2) $$x_{i}^{\prime }=x_{i}+m_{0}+m_{x}\Delta x+m_{y}\Delta y+\frac{1}{2}m_{xx}\Delta x^{2}+\frac{1}{2}m_{yy}\Delta y^{2}+m_{xy}\Delta x\Delta y+\ldots \;y_{i}^{\prime }=y_{i}+n_{0}+n_{x}\Delta x+n_{y}\Delta y+\frac{1}{2}n_{xx}\Delta x^{2}+\frac{1}{2}n_{yy}\Delta y^{2}+n_{xy}\Delta x\Delta y+\ldots$$

The zero-order term $\left(m_{0},n_{0}\right)$ describes a rigid body translation of the subset. The first-order term simulates the linear deformation of the subset (rotation, shear, etc.), while the higher-order term is sometimes used to simulate more complex nonlinear deformations. The calculation result is shown in Figure 1d.

The calculation of the wavefront gradient is the ratio of this translation to the distance between the crystal and the detector. Since the angle *α* is associated with the phase gradient ∇ϕ [29], we can use the following equation:

(3) $$\nabla \phi \left(x,y\right)=\frac{2\pi }{\lambda }\alpha_{x,y}\approx \frac{2\pi }{\lambda }\frac{vs}{L}$$

where *λ* is the photon wavelength, and *L* is the distance between the crystal and the detector. The model assumes that the speckle pattern has a rigid translation and does not consider the distortion.

This method can be understood as a high spatial frequency intensity modulation of the wavefront, which uses stationary speckle to track the geometric path of light passing through each pixel of the detector. Each image subset contains a different speckle pattern. Furthermore, the speckle pattern acts as a single marker and can be digitally tracked between images taken on different planes in space. The size of the image subset is selected according to the required resolution and signal sensitivity. In most cases, the data recorded by two-dimensional scanning is the projection of v, and then two orthogonal transverse phase gradient components can be extracted. One-dimensional scans with fewer images have also proved to be an effective method to restore a one-dimensional phase gradient. Since the vertical plane is the tangential plane of the beam deflection optical system, the calculation is only carried out in the vertical plane.

In most speckle-based techniques, the correlation parameter is the transverse correlation length that can be predicted by the Van-Cittert theorem [30]. It is important to understand the coherence length for speckle pattern processing, whether as an information carrier or to suppress the coherence length when the effect is regarded as noise. The transverse coherence of the beam is the most important factor affecting the speckle observation, and the maximum distance between the observed scattering characteristics and the interference pattern is set [31]. However, the transverse coherence of a few microns is sufficient to generate a usable speckle pattern. A typical transverse coherence length at a sample position for *E* = 15 keV at the Shanghai Synchrotron Radiation Facility (SSRF) 09B is $l_{t}\approx \frac{\lambda z}{2f_{s}}=25$ µm, where z is the distance from the source, and $f_{s}$ is the full width half maximum (FWHM) size of the source.

The detector response function also leads to the degradation of the quality and clarity of the visible speckle pattern. In fact, it acts as a low-pass filter, blurring and smoothing the high frequencies. In order to obtain good contrast in speckle patterns, it is important to adjust the size of speckle particles to the resolution of the detector. X-ray detectors are divided into the integrating type and the counting type. A more in-depth explanation of these differences and their effects on X-ray signal measurement can be found in [32]. For our experiments, two-dimensional high spatial resolution is a decisive requirement. Charge-coupled device (CCD)-based two-dimensional integrator imaging detectors coupled to scintillator and microscope optics are our preferred detectors. For X-rays, these chips are not directly illuminated but are connected to a thin scintillator that converts the X-ray intensity into visible light and then records the image on a CCD through a visible light microscope with a certain magnification. Due to the use of CCD technology, these cameras must be combined with the shutter to avoid artifacts when the chip simultaneously collects light and reads the electronic level. The camera has optical aberrations, since visible light is used to obtain high magnification. The recorded images present distortions, and in some cases compensation must be made. To describe and correct this unnecessary effect, the distortion of the imaging system can be calculated using the XST technique. The idea is that two images are collected with the same speckle position, but the detector is shifted by a small amount of δ. Therefore, the expected transformation between images is a rigid translation of all subsets, and for a distortion-free detector, we have ν=δ. The local distortion of the detector is very small (less than 1% even at the corner), and the maximum amplitude of the displacement vector ν is several pixels. In the absolute metrology mode, the subsets of two images corresponding to two speckle sets are located in the detector field of view region close to each other, and the error caused by the detector distortion can be ignored. The common method to improve the contrast of the speckle image and reduce the nonuniformity of the beam is to deduct the noise of the detector and the spatial fluctuation of the incident beam. The intensity of the speckle pattern recorded by the detector can be adjusted to

(4) $$I_{r}=\frac{I_{0}-I_{dark}}{I_{beam}-I_{dark}}$$

where $I_{0}$ is the original speckle patterns recorded by the detector, $I_{dark}$ is an average of several acquisitions with the beam shutter closed, and $I_{beam}$ is the flat-field image acquired without the scattering object.

The attainable angular sensitive depends directly on the scanning step *s* and on the working distance L between the crystal and the detector. The smallest deviation that can be measured is given by $\vartheta_{sensitive}=s\times \delta_{c}/L$, where $\delta_{c}$ is the pixel accuracy of the cross-correlation criterion [27]. In our experimental setup, $\delta_{c}\leq 0.01$ pixel, L=1.1 m, and s=2 µm, leading to a theoretical accuracy of $\vartheta_{sensitive}$ ~ 19 nrad. Using a smaller scanning size or placing the detector further away from the membrane would result in a higher sensitivity.

## 3. Experiment and Results

### 3.1. Experiment

The at-wavelength characterization of five channel-cut crystals was determined experimentally by the above method. The experiment was carried out in the Test beamline 09B of the SSRF, which is dedicated to the development of optics, metrology, and instrumentation [33]. The photon energy of 15 keV was selected using a double crystal Si-111 monochromator with a bandwidth of ΔE/E ~10^−4^. The channel cut under the test was mounted on the high precision turntable and placed 40.3 m from the X-ray source. A Flash 4.0 CCD camera coupled to a scintillator was mounted on a motorized stage and located at 1.1 m further downstream the crystal. The effective pixel size of the imaging system was *p* = 0.65 µm and *p* = 5.2 µm (only on X-ray crystal morphology of CC1). An abrasive paper (FEPA Grit) with an average pore size of 5 µm and thickness of about 150 µm, which works nicely in the hard X-ray energy range, was mounted on a piezo stage and placed 0.39 m upstream from the crystal. The beam size was defined using slits located downstream of the monochromator to only illuminate the region of the channel cut within the camera field of view. Two groups of images were recorded: with (Crystal Speckle) and without the channel cut (reference speckle) in the X-ray beam. A total of 102 images were obtained by scanning the sandpaper, which crosscut the X-ray beam with a step size of s = 2 µm.

### 3.2. Channel-Cut Crystal Preparation Processing

To verify the effect of different crystal processing methods on the wavefront measurement results, five crystals were prepared. Firstly, the crystal was cut by two different cutting methods, namely cylindrical cutting and diamond wire cutting. The cylindrical cutter is manufactured from welded cemented carbide and is suitable for side milling of large workpieces. It has good rigidity, better chip removal, excellent cutting ability, and a high metal cutting rate, which allows it to mill quickly and avoid breakage during heavy milling. The diamond wire saw cutter uses cutting by abrasion rather than by saw teeth. Due to the hardness of diamonds, the abrasion technique can be used to cut almost any material softer than the diamond abrasive. Compared with the traditional solid blade, the process also produces fewer kerf and wasted materials. A diamond wire saw is an environmentally friendly, highly efficient, and safe cutting tool, which is especially used for cutting and separating crystal materials, magnetic materials, or sapphire chips. Its working principle is to grind and cut the object with a wire saw under a certain tension, remove the debris and heat with cooling water, and finally divide the silicon via squaring and slicing into several parts.

**Figure 2:**
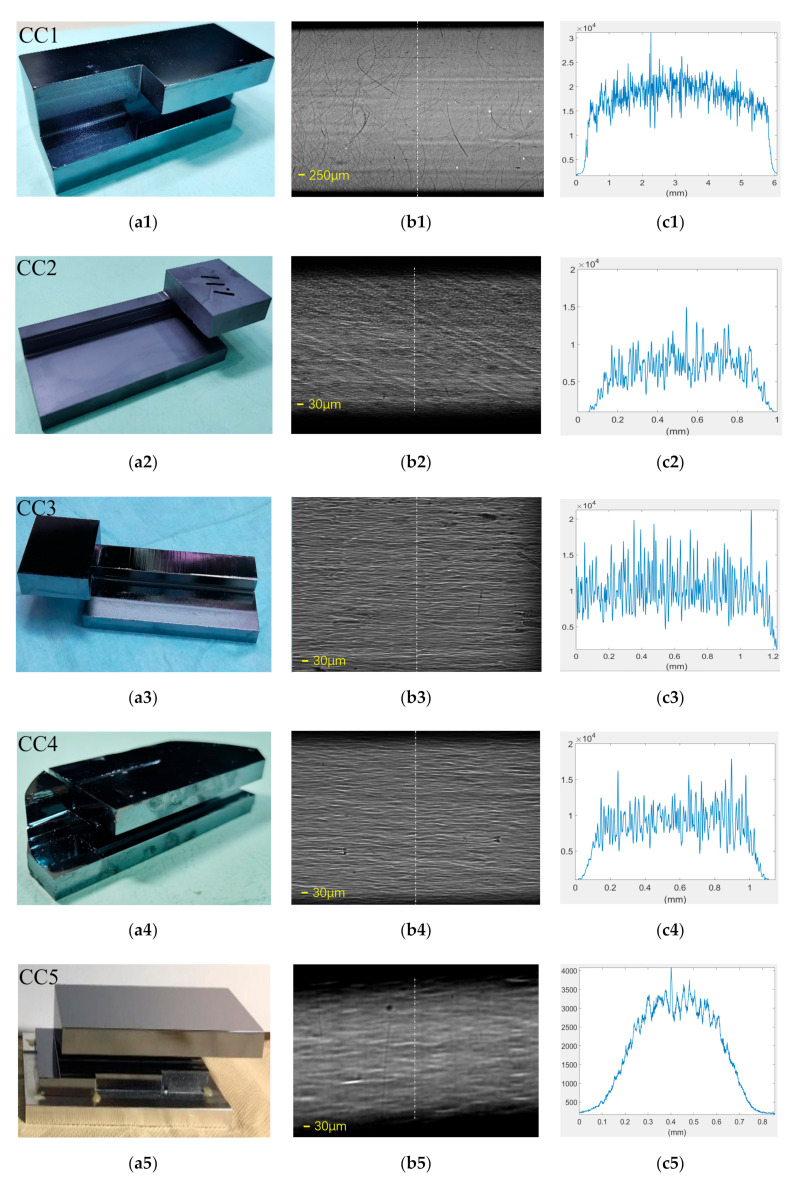
(**a1**–**a5**) Photos of channel-cut crystals from CC1 to CC5. (**b1**–**b5**) The X-ray crystal morphology of five channel-cut crystals, and (**c1**–**c5**) the intensity profiles taken at the dashed lines.

When the crystal cutting is finished, the important work of chemical corrosion is then applied. The required removal thickness of each crystal is different and must be decided according to actual working conditions. The corrosive agent used in the process of chemical corrosion of a crystal is a mixed solution of 95% concentrated nitric acid and 45% hydrofluoric acid. When preparing this mixed solution, the concentrated nitric acid and hydrofluoric acid should be mixed in a fixed proportion (volume ratio HNO_3_:HF = 10:1) with a measuring cylinder and then poured into a customized Teflon tank. It should be noted that the capacity of the etchant should be sufficient to ensure complete immersion of the crystal within it. A total of five channel-cut crystals was measured in this study. Two of them were purchased from different commercial companies, and the other three were made by us. Photos of the final tested channel cut are shown in Figure 2a. The channel-cut CC1 was bought from Crystal Scientific (UK) Ltd. Using the Si-111 diffraction surface, the Bragg angle was 7.575° at the photon energy of 15 keV. The orientation accuracy of working face was X: 0.13° and Y: 0.2°. The surface of the working face was polished by the mechanical-chemical method, and the roughness was about 0.5 nm. The CC2 was also an Si-111 crystal and was given by Photon Factory (PF) in Japan. Unfortunately, the detailed production process of this crystal is not clear. However, the crystal morphology was very distinctive and representative and is discussed in the results section.

CC3 to CC5 were made by the Institute of High Energy Physics, Chinese Academy of Sciences, Beijing. Their processing processes are as follows:

For CC3, a 6-inch zone melting single crystal silicon rod was selected with an axial <111> (impurity concentration of raw material was 10^−11^), and the channel cut crystal of the Si-111 working face was processed by cylindrical cutting equipment. The orientation accuracy of the working face was X: 0.05°and Y: 0.15°, and the working face width was 20 mm. The crystal was then immersed in the corrosion tank for corrosion, ensuring that the whole crystal was immersed in the corrosive agent. The process of chemical corrosion was about 12 min. After the corrosion, the crystal was taken out, and the thickness stress layer of the crystal removed was about 40 μm.

CC4 was made by a solar grade single crystal silicon rod (impurity concentration 10^−6^), which was also processed by cylindrical cutting equipment. Compared with the above-mentioned high-quality silicon single crystal, the impurity concentration of the solar grade single crystal silicon rod was several orders of magnitude higher. A channel cut with a working face of Si-111 was prepared using a solar grade single crystal silicon rod in order to test the influence of high impurity concentration on the beam quality. The orientation accuracy of the working face was X: 0.13° and Y: 0.2°. The corrosion time was about 20 min, and the removal amount of the stress layer was about 100 μm.

CC5 was the only crystal used with an Si-220 diffraction surface. It was prepared on the side face of a 6-inch zone melting silicon rod with axial <111> (impurity concentration of raw material was 10^−11^). As opposed to CC3 and CC4, it was machined by diamond wire cutting. The orientation accuracy of the working face was X: 0.15° and Y: 0.25°. Chemical corrosion with the same corrosive agent was used in this channel cut. In order to achieve removal of a 500 μm thickness stress layer, the chemical corrosion time was increased to about 50 min. The preparation parameters of all five crystals are shown in Table 1.

### 3.3. Crystal Morphology

Figure 2b shows the X-ray crystal morphology of the five tested channel cut crystals in this study. To obtain a better signal-to-noise ratio (SNR) in an atmospheric environment, the photon energy was selected to be 15 keV, where the Bragg angle was 7.575° for the Si-111 crystal and 12.43° for the Si-220 crystal. The X-ray morphology in Figure 2b shows the different traces left by different processing technology. The surface of CC1 was very flat with almost no trace of cutting trajectory left except some deep and small scratches on the surface. The width of the deep scratch was about 12 pixels (62 μm), which would cause a 60% decrease in intensity. Correspondingly, the width of the shallow scratch was about 6 pixels (31 μm), which would cause a 40% decrease in intensity. It should be pointed out here that due to the relatively large size of the CC1 crystal, a 1.25× *g* magnification conversion system was chosen in the experiment to observe as large an area of the beam spot as possible; consequently, the effective pixel size of the CCD camera was 5.2 μm. However, the other four crystals did not need to cover such a large area in the actual environment, so the crystal size was smaller. A 10× magnification conversion system was applied in the experiment, corresponding to an effective pixel size of 0.65 μm of the CCD camera. The exposure time of all crystal morphologies was the same and was chosen to be 10 s in order to guarantee the same experimental conditions. The intensity profiles taken in the dashed lines area are shown in Figure 2c. Although the intensity curve of CC1 (as shown in Figure 2c1) was relatively high, this was due to the efficiency of magnifying the lens.

The case of the CC2 surface was worse, since an oblique cutting trajectory could be clearly observed. Although the specific preparation process was not clear, the trace on the surface was likely left by the blade during cutting. Worse still, this channel cut was not well polished or corroded after cutting. Such a surface would have a great impact on follow-up experiments.

The X-ray morphology of CC3 and CC4 looked very similar because the same cutting process and corrosion methods were adopted in these two channel cuts. The surfaces of them were irregular, which was likely the result of corrosion. The effect of chemical corrosion caused a continuous trace on the X-ray intensity, and it was shown as the appearance of a wave. The doping concentration of these two crystals was different, since CC4 was made using a solar grade single crystal silicon rod, while CC3 was selected by melting a single crystal silicon rod. However, the X-ray diffraction efficiency of these two crystals was almost the same (as shown in Figure 2(c3,c4)).

CC5 used diamond cutting, and the corrosion time was the longest, which made its morphology look much smoother. The beam spot of CC5 was slightly inclined, and the reflectivity was much lower than the others because its diffraction plane was Si-220.

### 3.4. Wavefront Measurement Results

**Figure 3:**
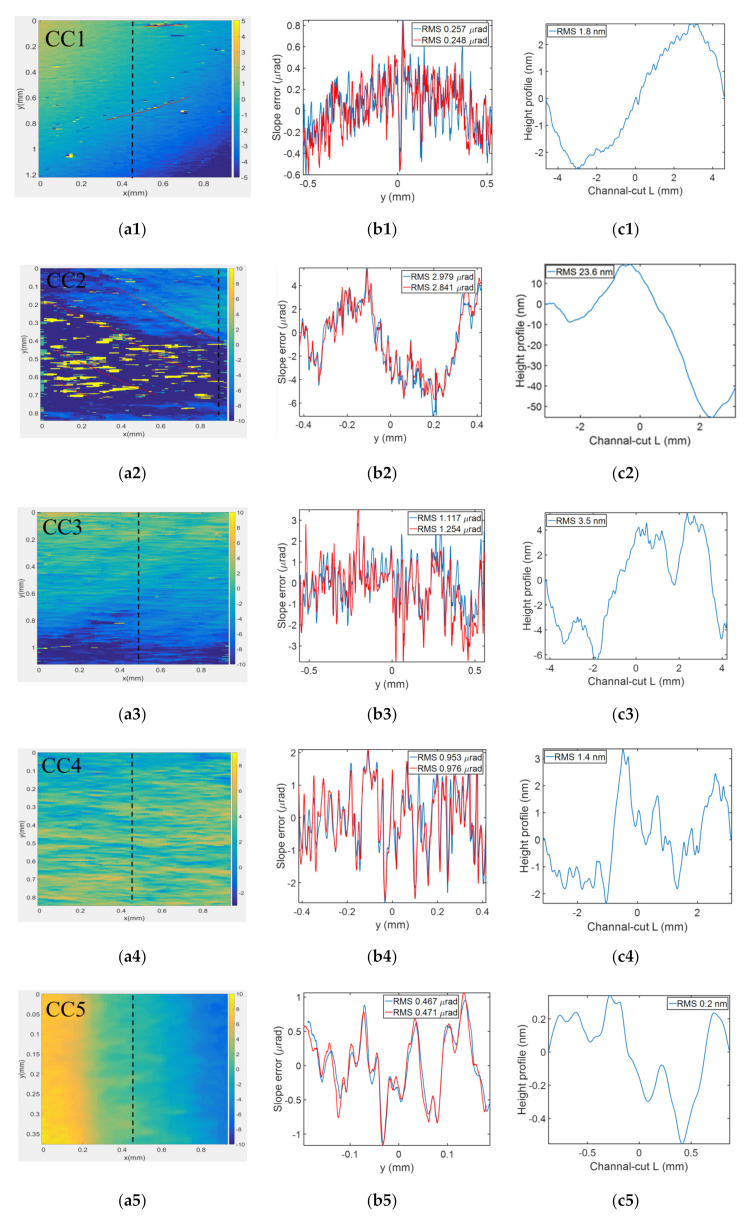
(**a1**–**a5**) The vertical wavefront slope errors, and (**b1**–**b5**) slope errors corresponding to the black dashed line. (**c1**–**c5**) The residual height profile of slope errors in the meridional direction for five channel cuts.

The vertical wavefront slope errors, measured using the speckle scanning technique, are shown in Figure 3a. The region of interest (ROI) of the images was different, since the size of each channel cut was different. The goal was to illuminate the target surface of CCD as much as possible. The beam size was about 8 mm (vertical) in the crystal surface with a Bragg angle of 7.575° (Si-111 crystals), and 1.8 mm (vertical) with a Bragg angle of 12.43° (Si-220 crystals). The horizontal beam size was 0.97 mm, corresponding to 1500 pixels. The scratch of CC1 and the oblique cutting trace of CC2 led to many poor points in the wavefront slope errors, such as the yellow bright spot in the figures. Even the direction of the scratch could be seen in the diagram (indicated by a red line in the figure), and CC2 was especially serious. These traces were too deep, resulting in the loss of wavefront information carried by the pixels. The line profiles in vertical directions (corresponding to the black dash line in the wavefront images) with different channel cuts are shown in Figure 3b. To avoid poor areas, the right area in CC2 was selected to extract the slope error curve. The local oscillation of these slope curves was relatively large, and there were two ways to analyze the problem. One was to use different parts of sandpaper to form speckles and compare the results of two measurements to eliminate random noise. We measured the five channel cut crystals each twice and compared the slope curves of the same line, as shown in the red and lake blue lines in Figure 3b. The results of the two measurements showed that the curves of the same channel cut were in good agreement, except for high-frequency information, and the RMS of these lines were almost the same. The vertical RMS slope errors were 0.26, 2.98, 1.25, 0.98, and 0.47 µrad from CC1 to CC5, which means the high-frequency jitter of the curve came from measurement error. The reason may be that the inhomogeneity of the crystal surface affected the SNR of the speckle. Figure 3c gives the residual height profile, which is related to the wavefront phase by h = ϕ/tan θ, where θ is the beam incidence angle on the crystal. The height profiles of CC1 and CC2 were consistent with a sine wave. However, the profile of the other three crystals made by us was random. They were not sine, cosine, or arc-shaped, and it may not have been possible to offset the effect on the wavefront by subsequent compensation. A summary of these five channel-cut crystal wavefront measurement results is shown in Table 2.

**Figure 4:**
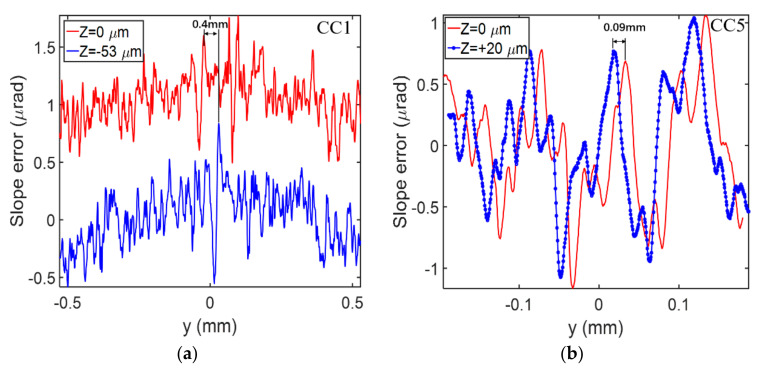
(**a**) The slope errors of different CC1 vertical positions. For comparison, the red curve is shifted up by 1 µrad. (**b**) The slope errors of different CC5 vertical positions.

The second method was to move the crystal vertically and observe the translation of the measurement results. Two representative channel-cut crystals (CC1 and CC5) were selected for the moving test. The measurement results are shown in Figure 4. The CC1 crystal was moved down 53 µm, and the corresponding spot on the crystal surface shifted backward about 0.4 mm. To facilitate observation, we shifted one of the curves upward by 1 µrad. As can be seen from Figure 4a, most of the high frequencies could not be overlapped. The CC5 crystal was moved up 20 µm, and the spot on the crystal surface shifted forward about 0.09 mm. Whether from different speckle positions or crystal movement measurement results, the slope errors of CC5 were in good agreement, indicating that these characteristics were carried by the channel cut itself. It can be considered that the results of corrosion affected the distribution of the wavefront curvature. Therefore, the speckle-based technique allowed us to draw conclusions on the surface quality of the crystal.

### 3.5. Simulation

To reduce the wavefront error caused by these channel cuts is to give full exploration of the advanced light, like the high energy photon source (HEPS) in China. Similar to other fourth generation sources, the HEPS has a multibend achromat (MBA) storage ring [34]. The specifications and the performance of the ring are shown in Table 3. This ring provides lower-emittance electron beams, resulting in more coherent X-ray beams with high brilliance. In addition to the innovation of the ring, the long insert device with many period numbers is widely used in order to determine the limits of its performance. In this simulation, we considered the insert device for the coherence beamline B4. The performance of the source is shown in Table 4. The source size was calculated using the Tanaka method. For all simulations presented, we selected the configuration using a single undulator CPMU 18 placed in the center of the straight section and tuned to its third harmonic at a photon energy of *E* = 12.4 keV.

**Figure 5:**
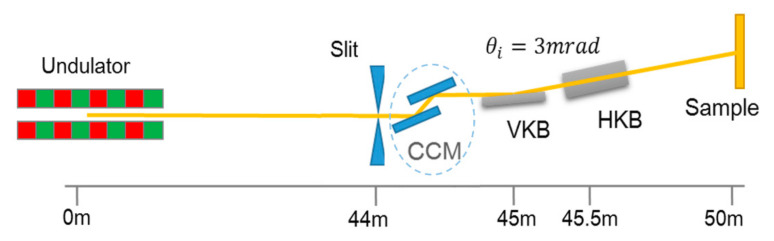
The layout of the beamline: VKB (vertical Kirkpatrick-Baez (KB) mirror) and HKB (horizontal KB mirror).

Referring to Shi’s work in advanced photon source (APS) [35], the requirement of the different experiments on the quality of the X-ray beam changed. For the diffraction-limited experiment, the coherence of the beam was very important for the size of the focusing spot. For the magnification, the uniform distribution and high flux of the spot were of concern. In the following simulation, we considered two cases with acceptance angles of 5 and 15 μrad, which were equal to the 1σ and 3σ of the beam divergence, respectively. For a small acceptance angle, the collected light was almost completely coherent. The layout of the beamline is shown in Figure 5. The direct focusing scheme using a Kirkpatrick-Baez (KB) mirror was considered. The vertical KB mirror (VKB) and horizontal KB mirror (HKB) were placed at 45 and 45.5 m, respectively, and the light was focused on the sample at 50 m. The magnification, M, of the system was 9.0 and 10.1 for VKB and HKB, respectively. The deflection of the monochromator changed as required by the beamline features. Therefore, the measured wavefront data were added to VKB and HKB instead of the modeling monochromator in the beamline. However, the effect on the beam spot was the same.

The simulation was carried out using the XRT development tool at MAX lab. Since the coherence in the advanced source was improved significantly, the wave propagation method for modeling the beam transport was necessary for this application. The electron number was chosen as 3000 to ensure the accuracy of the simulation and balance calculation time. In addition, the experiments were also limited to the monochromatic beam in order to illustrate the wavefront error issues clearly without considering the thermal deformation. CC2 and CC3 were not considered because the wavefront distortion was too large.

(1) Demagnification dominated case

**Figure 6:**
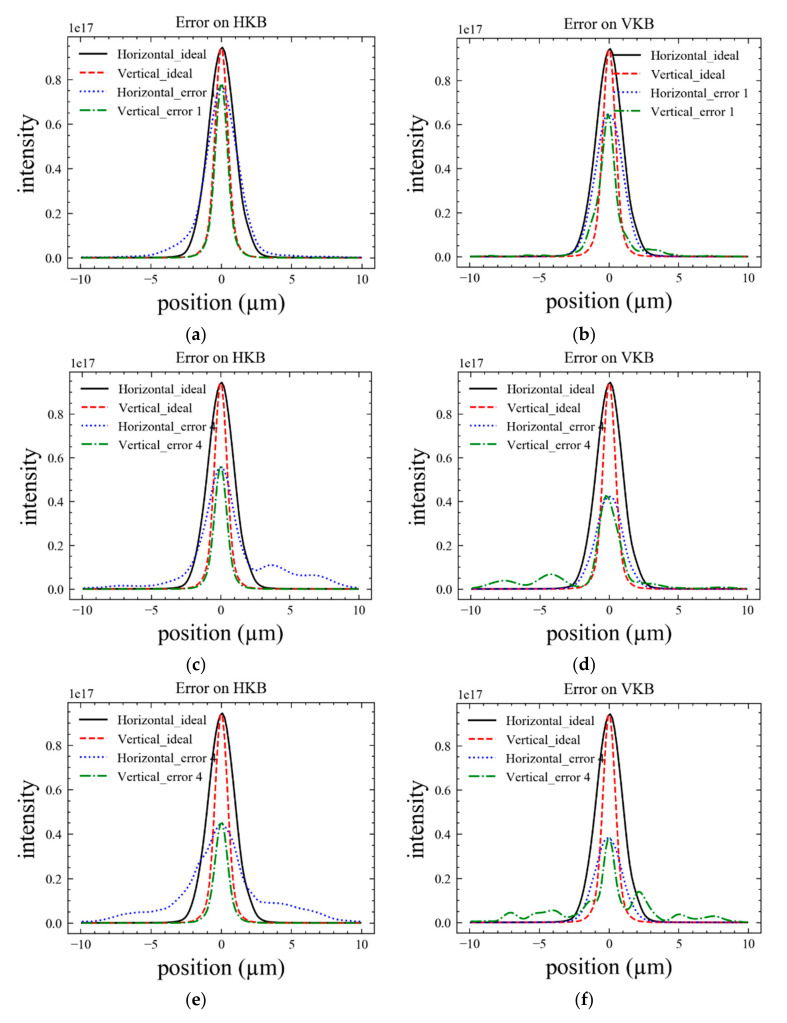
The plots of the intensity distribution with 15 μrad acceptance angle for (**a**,**b**) CC1, (**c**,**d**) CC4, and (**e**,**f**) CC5. The error was added separately for (**a**,**c**,**e**); no error on VKB.

The X-ray transported to the sample was partial coherence in this case. Figure 6 shows the simulation 1D shape of the intensity distribution for different measured errors. In each picture, the solid line is the ideal result. The left column corresponds to the case with the error on the HKB, and the right one corresponds to the VKB mirror. The Strehl ratio (SR) and the width of the spot are summarized in Table 5. The low SR value for CC4 verified that the space distribution of the photons tended to disperse, which was not good for the experiment, due to its low flux density or low signal-to-noise. The width of the spot became indistinguishable when the wavefront distorted seriously. The uniform shape of the CC1 spot made it suitable for most of the experiments not requiring coherence. Another result from the simulation is that the error in the vertical direction caused lower SR than that in the horizontal direction. The horizontal deflection of the monochromator is a good choice when uniformity is required in the light field.

(2) Diffraction-limited case

**Figure 7:**
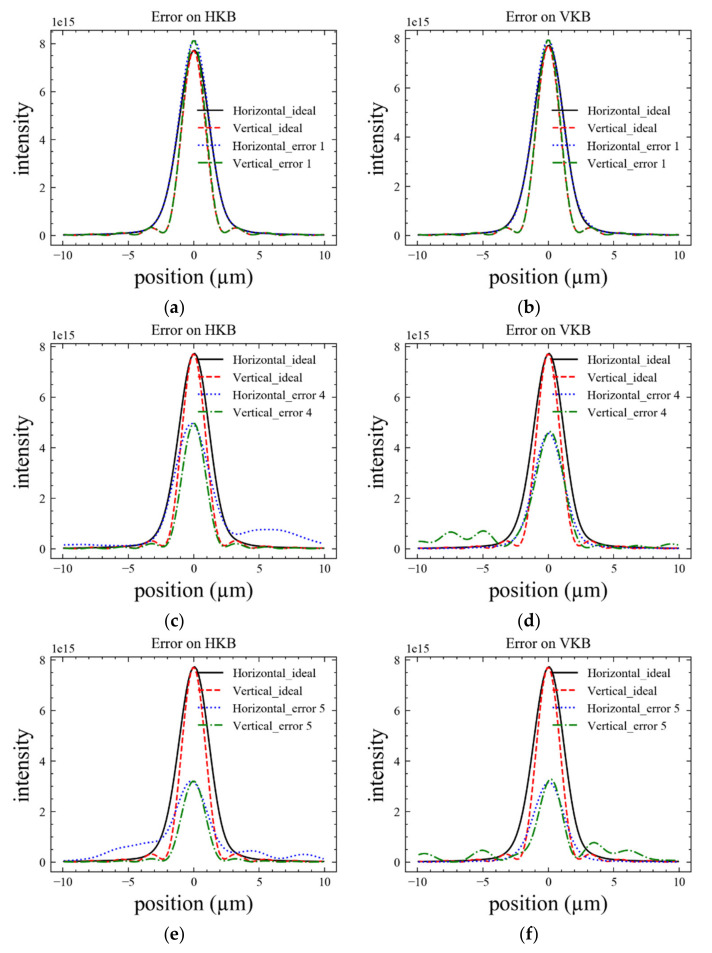
The plots of the intensity distribution with 5 μrad acceptance angle for (**a**,**b**) CC1, (**c**,**d**) CC4, and (**e**,**f**) CC5. The error was added separately for (**a**,**c**,**e**); no error on VKB.

A small acceptance angle means that only part of the wavefront contributed to the final spot compared to the demagnification case. The coherence fraction of the X-ray at the sample also increased, which was very useful for the coherent experiment method. In this case, the SR value can be computed by

(5) $$\mathrm{SR}=\exp\left[-{\left(\frac{4\pi }{\lambda }\sin\theta_{i}\right)}^{2}\sigma_{w}^{2}\right]=\exp\left(-\sigma^{2}\right)$$

where $\sigma_{w}$ is the surface height error, and σ is the introduced phase error. The results are shown in Figure 7 and Table 6. For each channel cut, the difference between the SR value by the vertical and horizontal error was small. The CC4 and CC5 with low quality had similar results as the partial coherence case. The deviation between the measured and theoretical SR value (about 0.97) for CC1 came from the simulation precision.

## 4. Conclusions

In summary, we applied the speckle-based scanning technique on diffraction crystals for the first time and demonstrated its potential using channel-cut crystals. The experimental results showed that different cutting and etching processes have a great influence on the crystal wavefront. The RMS values of slope errors ranged from 0.25 to 2.98 µrad. By increasing the etching time, the slope error of diffraction wavefront could be reduced. This was observed in the X-ray topography and the results of speckle wavefront measurement. In addition, we also used these wavefront errors to simulate the beamline’s efficiency. The simulation results showed that the low SR value verified that the space distribution of the photons tended to disperse, which was not good for the experiment for its low flux density or low signal-to-noise. The width of the spot became indistinguishable when the wavefront distorted seriously. Furthermore, the uniform shape of the spot makes it suitable for most experiments that do not require coherence. This technique should be of interest for those measuring the effects of crystal diffraction on the beam quality and will lead to further improvements in the fabrication of new X-ray crystals and their optimization on X-ray synchrotron beamlines.

## Figures and Tables

**Table 1:** Summary of five channel cut crystals’ preparation processing.

Channel-Cut	Diffractive Surface	Cutting Method	Thickness Stress Layer Removed (μm)
CC1	Si-111	Unclear	Unclear
CC2	Si-111	Unclear	Unclear
CC3	Si-111	Cylindrical cutting	40
CC4	Si-111	Cylindrical cutting	100
CC5	Si-220	Diamond wire cutting	500

**Table 2:** Summary of five channel cut crystals’ wavefront measurement results.

Channel-Cut	Wavefront Slope Error (μrad) RMS	Height Profile (nm) RMS
CC1	0.257 0.248	1.8
CC2	2.979 2.841	23.6
CC3	1.117 1.254	3.5
CC4	0.953 0.976	1.4
CC5	0.467 0.471	0.2

**Table 3:** Basic simulation parameters of 6 GeV storage ring and typical undulator in beamline for the high energy photon source (HEPS).

Storage Ring	Specification
Storage ring energy	6 GeV
Nominal beam current	0.2 A
Electron beam emittance	34.2 pm.rad (H)
Coupling constant	0.1
Relative energy spread values	0.00111
Electron beam sizes	8.8 μm (H) × 2.3 μm (V)
Electron beam divergences	3.1 μrad (H) × 1.2 μrad (V)
Insertion device	
Number of periods	234
Period length	17.9 mm
Minimum gap	5 mm
K-value at minimum gap	2.194

**Table 4:** Source characteristic photon beam size and divergence for selected B4 beamline positions.

Items	Value
Single electron radiation sizes RMS	4.61 μm
Single electron radiation divergences RMS	3.46 μrad
Source size RMS	9.97 μm (H) × 5.22 μm (V)
Source divergence	5.49 μrad (H) × 4.69 μrad (V)
Coherent fraction	0.29 (H) × 0.65 (V)

**Table 5:** The summarized performance of the simulated beamline with 15 μrad acceptance angle.

Chanel-Cut Crystal Error Data	Slope Error (μrad) RMS ^1^	Strehl Ratio	Width (μm, H × V) FWHM
Ideal	-	1	2.18 × 1.10
Horizontal CC1	0.19	0.82	2.66 × 1.10
Vertical CC1	0.19	0.68	2.34 × 1.10
Horizontal CC4	0.97	0.61	2.50 × 0.94
Vertical CC4	0.97	0.44	2.18 × 1.10
Horizontal CC5	0.47	0.47	3.44 × 1.24
Vertical CC5	0.47	0.40	2.34 × 0.94

^1^ The RMS of slope error in the range of the simulated beam size.

**Table 6:** The summarized performance of the simulated beamline with 5 μrad acceptance angle.

Chanel-Cut Crystal Error Data	Slope Error (μrad) RMS ^1^	Strehl Ratio	Width (μm, H × V) FWHM
Ideal	-	1	2.82 × 2.18
Horizontal CC1	0.23	1.05	2.82 × 2.18
Vertical CC1	0.23	1.06	2.82 × 2.18
Horizontal CC4	1.13	0.64	3.12 × 2.18
Vertical CC4	1.13	0.59	2.66 × 2.50
Horizontal CC5	0.46	0.42	2.96 × 2.18
Vertical CC5	0.46	0.41	2.66 × 2.18

^1^ The RMS of slope error in the range of the simulated beam size.
